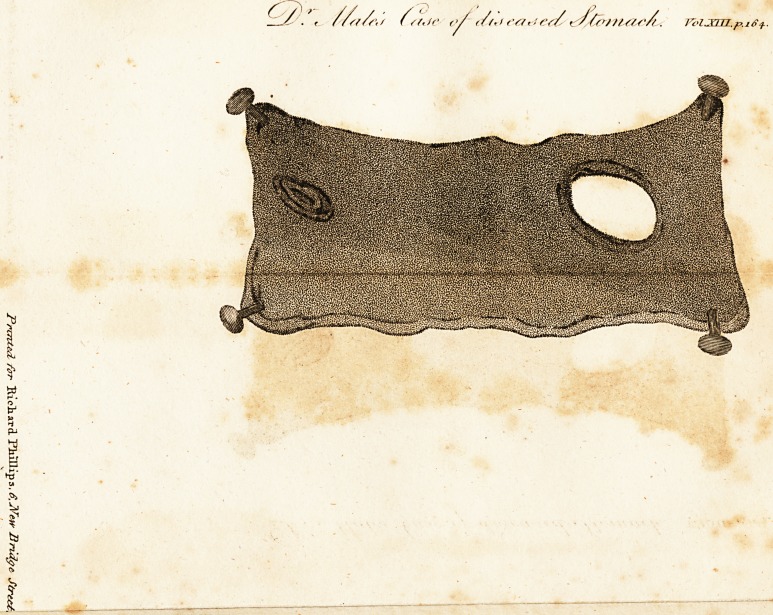# Extraordinary Appearance of the Stomach, Discovered on Dissection

**Published:** 1805-02-01

**Authors:** Geo. Edw. Male


### Extraordinary Appearance of the Stomach, *discovered on Dissection*; *by* Geo. Edw. Male, M. D.

Sept. 21, 1803, Mary Darlington, ætat. 15 years, had been complaining for two or three days of slight pain in her bowels, which became greatly aggravated on the morning of the 21st inst. I was called to her in the evening of the same day, and found her labouring under enteritis. Her countenance was pale and ghastly; pulse 150; urine scanty, and high coloured; body costive. She could give no account of the cause of her disease. I was informed she had had this complaint, accompanied with gastritis, about four years ago, since which time she had occasionally complained of a pain in her stomach. She had taken an emetic, and some gin with the tincture of rhubarb, by the advice of one of her female neighbours. I ordered ℥x of blood to be taken from her arm, and a glyster, with an ounce of castor oil, to be exhibited immediately. Her abdomen was directed to be fomented with a decoction of chamomile; and her thirst being extremely urgent, she was allowed toast and water made tepid to drink. At eleven o'clock I was informed no blood could be obtained by the lancet; leeches were applied to the abdomen, but as she was then evidently about to expire, nothing farther was done, and she died about an hour afterwards.

### *Appearances on Dissection.*

Leave having been obtained to inspect the body, it was opened on the morning of the 23d of September, thirty-six hours after death, by Mr. Edwards, Apothecary, at the Dispensary.

The omentum was found adhering very firmly to the peritoneum on both sides, and also to the intestines in several places. A very large quantity of serous fluid was discovered in the cavity of the abdomen, amounting to several quarts, with considerable effusion of coagulable lymph. The small intestines exhibited marks of general inflammation, and the larger ones were slightly affected and much distended with air, but no appearance of gangrene was discoverable. The liver was rather small, and of a paler colour than natural. The stomach was empty, and appeared partially inflamed; and in the superior part of it, near the cardia, was a foramen, nearly circular, about three quarters of an inch in diameter, perfectly smooth and regular; regular; it had not at all the appearance of having been eroded by the gastric juice, or of being the effect of *recent* inflammation. On the opposite side of the same organ was another foramen, nearly of an oval form, not quite half an inch in length, which however did not perforate the external coat of the stomach, but had apparently formerly done so, and afterwards become closed, and the edges united by a cicatrix. The other parts of the stomach were perfectly sound. I presented the stomach to Mr. Freer, surgeon, in Birmingham, who has preserved it in his collection. The rectum contained a few hardened scybala, bu the other parts of the intestinal canal were nearly emp ty. I have enclosed a rough sketch of the parts, of which y ou may make what use you please.

*Birmingham, Dec.* 8, 1804.

EXPLANATION OF THE PLATE.

a. A part of the interior side of the stomach, the other parts being cut away.

b. A foramen about half an inch in length, nearly oval, but not perforating the external coat.

c. A foramen in the superior surface of the stomach, about two inches in circumference.

## Figures and Tables

**Figure f1:**